# Platelet Number and Indexes during Acute Pancreatitis

**DOI:** 10.5005/ip-journals-10018-1104

**Published:** 2014-07-28

**Authors:** Ayse Kefeli, Sebahat Basyigit, Abdullah Özgür Yeniova, Metin Küçükazman, Yasar Nazligül, Bora Aktas

**Affiliations:** 1 Department of Gastroenterology, Kegibren Education and Research Hospital, Ankara, Turkey; 2 Department of Gastroenterology, Sinop State Hospital, Sinop, Turkey

**Keywords:** Acute pancreatitis, Mean platelet volume, Platelet count.

## Abstract

**Aim:**

Acute pancreatitis (AP) is an inflammatory disorder, the incidence of which has been increasing over recent years. Mean platelet volume (MPV) is an index of platelet activation and influenced by inflammation. The objective of the present study is to assess whether MPV would be convenient parameters for predictor factor of patients with AP.

**Materials and methods:**

A total of 140 AP patients (male/female: 63/77) and 70 healthy subjects (male/female: 23/47) were enrolled in this study. The following data were extracted from the hospital medical records, including age, sex, platelet count, MPV, were recorded at the time of admission and as well as at the 1st day of remission of the disease.

**Results:**

Mean platelet volume levels at onset and remission of AP were 7.8 ± 1.6 and 7.7 ± 0.9 respectively, and there was no statistically significant difference between these groups. Platelet count at onset and remission of AP and control subjects was 203 ± 74 × 10^3^/μl, 234 ± 76 × 10^3^/μl and 251 ± 87 × 10^3^/μl, respectively, and there was statistically significant difference between these groups. Platelet count at onset and remission of AP was statistically lower than control subjects.

**Conclusion:**

Some studies in literature suggest that MPV might be a useful parameter to be used as an indicator for AP and a prognostic factor for AP, but, in this study, it was revealed that MPV values do not change at AP compared with controls. Therefore, further prospective studies investigating the factors affecting the platelet size are required to determine whether MPV has a clinical implication and for predictor value of patients with AP.

**How to cite this article:** Kefeli A, Basyigit S, Yeniova AÖ, Küçükazman M, Nazligul Y, Aktas B. Platelet Number and Indexes during Acute Pancreatitis. Euroasian J Hepato-Gastroenterol 2014;4(2):67-69.

## INTRODUCTION

Acute pancreatitis (AP) is a common clinical condition; the incidence of which has been increasing over recent years.^1^ It is a disease of variable severity in which some patients experience mild, self-limited attacks, while others manifest a severe, highly morbid and frequently lethal attack. The exact mechanisms by which diverse etiological factors induce an attack are still unclear. Most cases are secondary to biliary disease or excess alcohol consumption.^2^ AP is an inflammatory disorder, which is characterized by a complex cascade of immunological events, which is not related pathogenesis but also bears importance in determining the course of disease. At present, it is widely accepted that the premature activation of digestive enzymes within the pancreatic acinar cells is a critical initiating event that leads to autodigestion of pancreas.^3^

Although radiological imaging modalities, pathologic and biochemical analysis are commonly used to monitor pancreatic inflammation, a great number of invasive/ noninvasive methods are also been studied for AP diagnosis and determining the disease activity. But, the role of platelets in the AP pathophysiology has not been clearly elucidated yet. In this setting, the main aim of the present study was to evaluate the platelet number and size alterations between onset and remission of the disease.

## MATERIALS AND METHODS

A retrospective review of the available records of 140 patients with AP admitted at the Kegioren Training and Research Hospital from January 2008 to September 2011 and with discharge of a diagnosis of AP was included in this study. Seventy healthy subjects were enrolled retrospectively into the study. Healthy controls recruited from the healthy adults without any history of acute/ chronic inflammatory disorders or history of usage of drugs.

Diagnosis of AP was based on the presence of severe abdominal pain, tenderness in the mild epigastrium and serum amylase level three times higher than normal. The following data were extracted from the hospital medical records, including AP etiology, age, sex, radiologic imaging, and laboratory test at onset and remission of disease. Remission was considered by initial symptoms disappeared, the patient started to take oral nutrition and amylase returned to normal levels.

Platelet number, MPV were recorded at the time of admission as well as at the 1st day of remission of the disease.

Exclusion criteria can be summarized as impaired pancreatic function (e.g. due to chronic pancreatitis or pancreatic carcinoma) and impaired platelet function, heart failure, acute or chronic inflammatory disorder, cancer and hepatic disease.

Data were analyzed by using a commercially available statistics software package (SPSS for Windows version 15.0, Chicago, Illinois, USA). Continuous variables were tested for normality by Kolmogorov-Smirnov test. Values were presented as mean ± standard deviation, in the case of non-normally distributed data, as median and range. Comparisons of percentages between different groups of patients were carried out using the chi-square test. Student’s t-test was performed for all normally distributed data. Mann-Whitney U-test was performed for normally distributed data for independent subgroups. Results are presented as mean ± SD, and p < 0.05 was regarded as statistically significant.

## RESULTS

About 140 patients with AP (63 males and 77 females) and 70 (23 males and 47 females) healthy control subjects were enrolled in this study. The mean ages of AP and control subjects were 57.9 ± 14.8 and 54.1 ± 16.1 years respectively. There was no significantly difference between the ages of the study participants.

The MPV levels at onset of AP and control subjects were 7.8 ± 1.6 and 7.8 ± 1.0 respectively, and there was no statistically significant difference between these groups (Table 1).

The MPV levels at onset and remission of AP 7.8 ± 1.6 and 7.7 ± 0.9 respectively, and there was no statistically significant difference between these groups (Table 1).

Platelet count at onset and remission of AP and control subjects was 203 ± 74 × 10^3^/μl, 234 ± 76 × 10^3^/μl, and 251 ± 87 × 10^3^/μl respectively, and there was statistically significant difference between these groups. Platelet count at onset and remission of AP was statistically lower than control subjects (Table 1).

**Table 1:** **Table 1:** Mean platelet volume (MPV) levels and platelet count at the onset and remission of acute pancreatitis and controls

		*Onset*		*Remission*		*Controls*		*p-value*	
MPV (fl)		7.8 ± 1.6		7.7 ± 0.9		7.8 ± 1.1		NS	
Platelet count (10^3^/μl)		203 ± 74		234 ± 76		251 ± 87		0.019	

p-value is for comparison between control and study population; NS: Not significant

## DISCUSSION

Acute pancreatitis is a systemic inflammatory process, which is often accompanied by thrombosis and bleeding disorders.^1^ The role of platelets in the pathophysiology of the disease has not been elucidated yet. It was found that both thrombocytopenia and thrombocytosis have been associated with pancreatitis.^4^ A study showed that an absence of thrombocytopenia (type 1) generally indicates a favorable prognosis, transient thrombocytopenia (type 2) also associates with a favorable prognosis, and persistent thrombocytopenia (type 3) generally indicate a poor prognosis.^5^ These results suggest that platelet count at admission and its change over the course of hospitaliza-tion can be useful measures for predicting prognosis in patients with AP. Two other studies have been assessed platelet count as a parameter for assessing the prognosis in AP have reported that the sequential organ failure assessment (SOFA) score, which enumerates the severity of organ failure, is useful for prognostic evaluation. Platelet count can be evaluated easily and promptly at admission in most clinical settings. Hence, assessment of platelet count is recommended from the standpoint of accuracy and convenience.^6,7^ In this study, it was revealed that platelet count significantly decreased at the onset and the remission of AP compared with controls, but there was not thrombocytopenia. Measurement of platelets over the course of hospitalization may be one of the most accurate and convenient parameters for precisely assessing the prognosis of patients with acute pancreatitis.

There are several studies indicating that the mean platelet levels would be associated with hypercoagula-bility in the course of AP were observed in the literature, but there are conflicting data. Three of these studies have reported that MPV, platelet distribution width (PDW) and platelet large cell ratio were decreased at onset of the diseases. Presence of large platelets and a significantly difference between onset and remission of the disease was documented in MPV.^8-10^ They suggested that platelets are directly involved in the systemic inflammatory process of AP, compensated by an immediate bone marrow response. The exact reason of decreased MPV in AP is not clear, but it is speculated that platelets not only control thrombosis and homeostasis but may also regulate inflammatory processes. Moreover, several cytokines that have been found to play a crucial role in the patho-genesis of AP may affect MPV. Studies show increased levels of tumor necrosis factor-α (TNF-α), interleukin-1 (IL-1), IL-6 and monocyte chemotactic protein-1 (MCP-1) in AP. Among these mediators, IL-6 is suggested to be the main factor responsible for the decreased levels of MPV.^11,12^ Another study showed that MPV levels elevated in AP compared with controls and MPV levels were still elevated when AP was in remission. They also found a positive correlation between MPV and pancreatic enzymes. Although, inflammation markers reduced at remission, MPV, D-dimer, fibrinogens were continuing to elevate. Indeed, among MPV, fibrinogen and D-dimer there were significantly positive correlations. Therefore, elevated MPV did not seem to be as a cause of acute inflammation in AP. Consequently, they suggest that MPV can reflect thrombotic status in AP.^13^ These studies showed that MPV were found changed in AP and suggest that MPV may be convenient parameters for predictor factor of patients with acute pancreatitis.

In this study, in contrast to other studies in the literature, it was revealed that MPV levels do not change at the onset and the remission of AP compared with controls.

## CONCLUSION

Some studies in literature suggest that MPV might be a useful parameter to be used as an indicator for AP. This parameter counted by clinical hematology analyzers is a simple, effortless diagnostic tool for platelet function and activation, and adds no extra cost or technical effort, but there are conflicting data about MPV values in AP. Therefore, further prospective studies investigating the factors affecting the platelet size are required, to determine whether MPV has a clinical implication and role in this disease.
